# Association between hemoglobin A1c and abdominal aortic calcification: results from the National Health and Nutrition Examination Survey 2013–2014

**DOI:** 10.1186/s12872-023-03700-2

**Published:** 2024-01-03

**Authors:** Can Cai, Lingsong Wang, Quanyao Chen, Min Lin, Xiuming Pan, Weida Chen, Danni Shi, Yao Chen

**Affiliations:** Scientific Research and Innovation Center, Women and Children’s Hospital, School of Medicine, Xiamen University, Xiamen, 361003 China

**Keywords:** Hemoglobin A1c, Abdominal aortic calcification, Cross-sectional study, NHANES

## Abstract

**Background:**

Hemoglobin A1c (HbA1c), a “gold standard” for the assessment of glycemic control, was associated with an increased risk of cardiovascular disease and coronary artery calcification. However, its effects on abdominal aortic calcification (AAC) are uncertain. The present study comprehensively investigated the association between HbA1c and AAC in the 2013–2014 National Health and Nutrition Examinations Surveys.

**Methods:**

Among 1,799 participants ≥ 40 years, dual-energy X-ray absorptiometry-derived AAC was quantified using the Kauppila score (AAC-24). Severe AAC was defined as a total AAC-24 > 6. Weighted linear regression models and logistic regression models were used to determine the effects of HbA1c on AAC. The restricted cubic spline model was used for the dose-response analysis.

**Results:**

The mean AAC-24 of participants was 1.3, and 6.7% of them suffered from severe AAC. Both AAC-24 and the prevalence of severe AAC increased with the higher tertile of HbA1c (*P* < 0.001). Elevated HbA1c levels would increase the AAC-24 (β = 0.73, 95% CI: 0.30–1.16) and the risk of severe AAC (OR = 1.63, 95% CI: 1.29–2.06), resulting in nearly linear dose-response relationships in all participants. However, this positive correlation were not statistically significant when participants with diabetes were excluded. Furthermore, subgroup analysis showed significant interactions effect between HbA1c and hypertension on severe AAC with the OR (95% CI) of 2.35 (1.62–3.40) for normotensives and 1.39 (1.09–1.79) for hypertensives (*P* for interaction = 0.022).

**Conclusion:**

Controlling HbA1c could reduce AAC scores and the risk of severe AAC. Glycemic management might be a component of strategies for preventing AAC among all participants, especially normotensives.

**Supplementary Information:**

The online version contains supplementary material available at 10.1186/s12872-023-03700-2.

## Background

Cardiovascular disease (CVD) is the leading cause of premature death and a major chronic disability for all regions of the world [1, 2]. Atherosclerosis, a manifestation of CVD, is characterized by several conditions including calcification within arteries [3]. These atherosclerotic calcifications are common in the abdominal aorta [4]. The prevalence of abdominal aortic calcification (AAC) was more than 85% in those aged over 65 years old [5]. Considering the universality of AAC and its prognostic value for CVD [6, 7], exploring its risk factors can contribute to complementing current evidence for CVD primary prevention.

Diabetes, a significant independent cardiovascular risk factor, contributes to the development of arterial calcification (AC) through multiple mechanisms [8]. The level of hemoglobin A1c (HbA1c) is a “gold standard” for the assessment of glycemic control [9]. Previous studies have investigated the effects of HbA1c on AC, especially coronary artery calcification (CAC). The cross-sectional and longitudinal study showed that higher HbA1c is independently associated with advanced CAC progression among individuals with or without diabetes [10, 11]. Previous studies have reported that CAC is rarely present without AAC, and AAC typically predates CAC [4]. Besides, AAC can be quickly and easily captured using low to negligible radiation exposure compared with assessing CAC [7]. Therefore, verifying the effects of HbA1c on AAC may complement existing primary prevention strategies for AC and future CVD.

The evidence on the correlation between HbA1c and AAC is limited. The Jackson Heart Study (JHS) have found that HbA1c was associated with the presence and extent of AAC among African Americans [12]. However, whether this relationships exist among other ethnic participants still needs to be investigated. Additionally, an analysis of 73 prospective studies (n = 294 998) showed that there was an approximately J-shaped association between HbA1c values and CVD risk [13]. However, a non-linear Mendelian randomization analysis in 373 571 white British participants from the UK Biobank indicated that the shape of the effect of genetically predicted HbA1c on cardiovascular outcomes was likely linear [14]. AAC is an independent risk of CVD, whether the shape of the effect of HbA1c on AAC is linear needs further research.

Therefore, the purposes of the current study were to assess the association between HbA1c and AAC among 1 799 participants ≥ 40 years of age from the National Health and Nutrition Examination Survey (NHANES). We hypothesized that elevated HbA1c was associated with increased risk of AAC and there was a linear dose-response relationship between them.

## Methods

### Study population

Data from the NHANES conducted by the National Center for Health Statistics (NCHS) was used in the current study. The detailed designs and protocols for the NHANES have been illustrated previously [15]. Briefly, it is an ongoing nationally-representative cross-sectional survey designed to monitor the health and nutritional status of adults and children across the United States using a stratified, multi-stage, and probability sampling method. The original data are publicly available at https://www.cdc.gov/nchs/nhanes/.

**Fig. 1:**
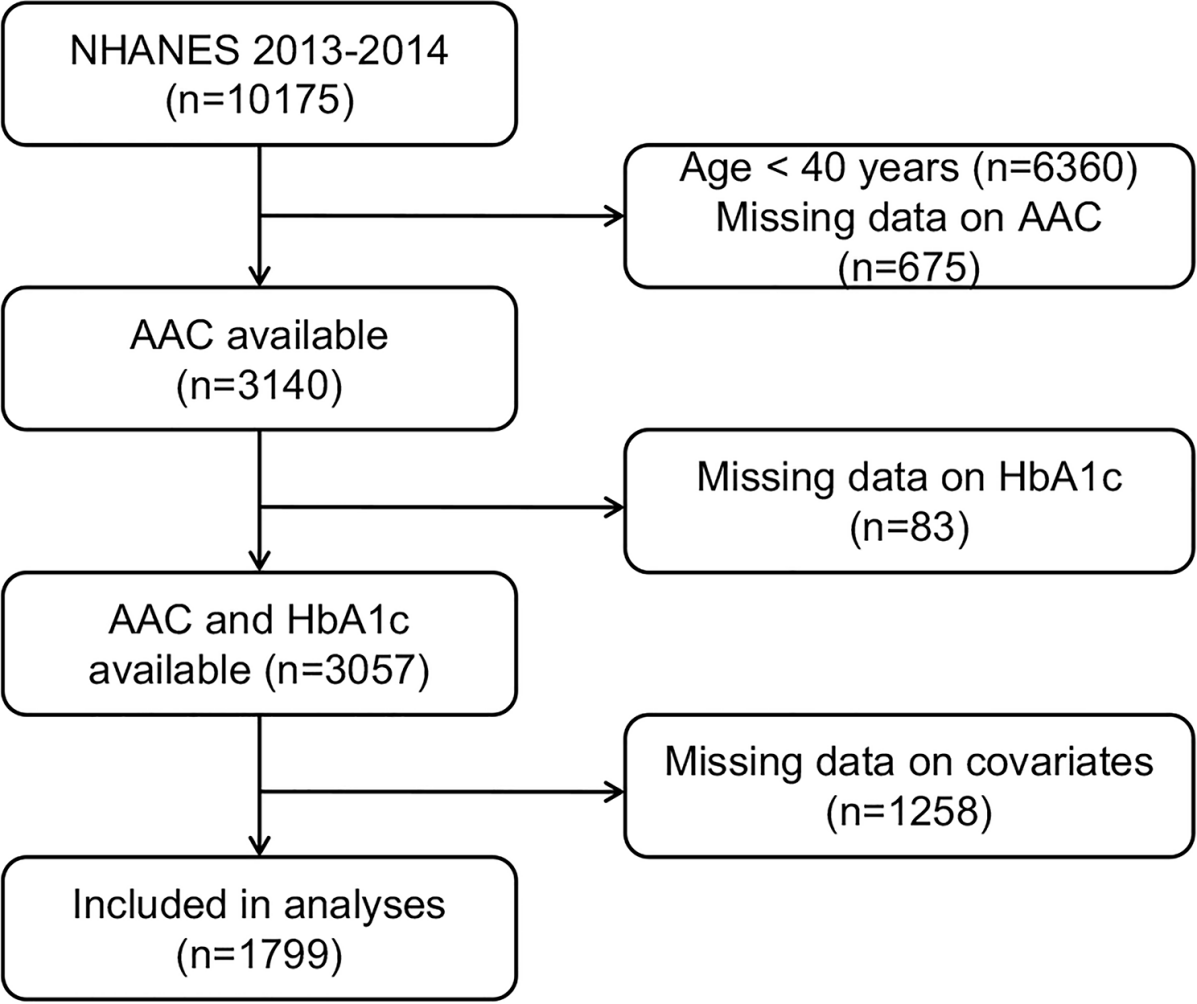
Flowchart of participant selection NHANES, National Health and Nutrition Examination Survey; AAC, abdominal aortic calcification, HbA1c, hemoglobin A1c

The current study was based on data from the 2013–2014 NHANES cycle since AAC status was only investigated in this survey cycle. In total, 10 175 participants were enrolled and 314 participants ≥ 40 years of age had available AAC data. Of these participants, we excluded 83 participants with missing data on HbA1c and 1 258 participants with missing data on covariates, such as demographics, lifestyle factors, examination data, and laboratory data. Finally, a total of 1 799 participants were available for the analyses (Fig. 1).

The NHANES was approved by the National Center for Health Statistics Research Ethics Review Board. Informed consent was obtained from each participant before the survey.

### Independent and outcome definitions

The independent variable was HbA1c levels in whole blood specimens. HbA1c, a percentage of the total amount of hemoglobin in the sample, was measured using the Tosoh Automated Glycohemoglobin Analyzer HLC-723G8, which applies non-porous ion exchange, high-performance liquid chromatography and microcomputer technology to obtain a quick and accurate measurement. The detailed laboratory procedure manual of blood sample collection and processing is publicly available at https://wwwn.cdc.gov/nchs/data/nhanes/2013-2014/labmethods/GHB_H_MET_GLYCOHEMOGLOBIN.pdf. HbA1c was analyzed as continuous and categorical variables, established based on the tertiles of HbA1c levels [HbA1c ≤ 5.4% (n = 659), 5.4%< HbA1c ≤ 5.8% (n = 546), and HbA1c > 5.8% (n = 594)]. Besides, since HbA1c ≥ 6.5% is one of the criteria for the diagnosis of diabetes according to the American Diabetes Association Professional Practice Committee [16], we also categorized participants into two groups [HbA1c < 6.5% (n = 1567) and HbA1c ≥ 6.5% (n = 232)] for further analysis.

The outcome variables were the AAC score and severe AAC. Detailed information about eligibility and exclusion criteria for measuring AAC is available at https://wwwn.cdc.gov/Nchs/Nhanes/2013-2014/DXXAAC_H.htm#DXXAL1CC. Briefly, AAC measurements were administered to eligible survey participants 40 years of age and older. Reasons for exclusion from the examination were as follows: (1) pregnancy; (2) self-reported history of radiographic contrast material (barium) use in past 7 days; (3) self-reported weight over 450 pounds; (4) other reasons including no time to complete the examination, pregnancy test not completed, and participant refusal, as well as exclusion for reasons other than pregnancy, such as a medical test. Besides, the main reasons for completed, but invalid, Instant Vertebral Assessment lateral spine scans were an insufficient scan area or partial scan, degenerative disease/severe scoliosis, sclerotic spine/spinal fusion/laminectomy, and poor image quality due to morbid obesity. Detailed information about the assessment is available at https://wwwn.cdc.gov/Nchs/Nhanes/2013-2014/DXXAAC_H.htm. Briefly, AAC was accurately recognized on lateral spine images obtained with dual-energy X-ray absorptiometry and the instant vertebral assessment lateral spine scans in the NHANES mobile examination center. Both AAC-24 and AAC-8 scoring semi-quantitative techniques (Kauppila score) were used for the evaluation [17, 18]. In the scoring method for AAC-24, the anterior and posterior aortic walls in front of the lumbar vertebrae L1-L4 were divided into eight segments. A score ranging from 0 to 3 was assigned within each part according to the calcific deposit proportions (0, no calcification; 1, one-third or less of the aortic wall; 2, more than one-third but less than two-thirds of the aortic wall; 3, more than two-thirds of the aortic wall), resulting in a range from 0 to 24 for the total score. Severe AAC was defined as a total AAC-24 score > 6 [19–21]. Besides, the AAC-8 scale, ranging from 0 to 8, estimated the total length of calcification of the anterior and posterior aortic walls in front of the same vertebrae separately (0, no calcification; 1, less than or equal to the height of one vertebra; 2, more than one but less than or equal to the heights of two vertebrae; 3, more than two but less than or equal to the heights of three vertebrae; 4, more than the height of three vertebrae). The AAC-8 score and severe AAC (AAC-8 ≥ 3) were the outcomes of the sensitivity analysis [22].

### Covariates

Potential covariates, including demographic characteristics [age, gender, race, education level, ratio of family income to poverty (RIP)], lifestyle risk factors [smoking status, alcohol drinking status, metabolic equivalent (MET)], physical examination information [body mass index (BMI), systolic blood pressure (SBP)], biochemical parameters [total cholesterol (TC)], estimated glomerular filtration rate (eGFR)], bone mineral metabolism markers (total 25-hydroxyvitamin D, serum calcium, serum phosphorus), and inflammatory indicator [neutrophil-lymphocyte ratio (NLR)] were selected based on previous studies [20, 23, 24]. The detailed measurement processes of these variables were available at https://www.cdc.gov/nchs/nhanes/. Briefly, demographic characteristics and lifestyle risk factors were collected using household interview questionnaires and mobile examination center (MEC) questionnaires. Additionally, physical examination information, biochemical parameters, bone mineral metabolism markers, and the inflammatory indicator were derived from medical examinations and laboratory assessments in the MEC.

BMI was calculated as weight in kilograms divided by height in square meters (kg/m^2^). A smoker was defined as someone who smoked at least 100 cigarettes during their lifetime. An alcohol drinker was defined as someone who drank more than 12 times per year. Physical activities were estimated as the aggregate weekly hours of moderate-to-vigorous activities multiplied by the MET levels, calculated using the following formula: MET-h/week = days× duration× MET levels. MET levels of moderate-intensity and vigorous-intensity physical activity were defined as 4.0 METs and 8.0 METs, respectively. Blood pressure (BP) was measured 4 times in the sitting position after at least a 5-min rest. The average BP readings were used in the analyses. Hypertension was defined as SBP ≥ 140 mm Hg or diastolic blood pressure (DBP) ≥ 90 mm Hg, using antihypertensive agents, or self-reported physician-diagnosed hypertension [25]. High cholesterol level was defined as having fasting TC ≥ 240 mg/dL or using lipid-lowing agents [25]. Diabetes was defined as having fasting plasma glucose (FPG) ≥ 126 mg/dL, a 2-hour plasma glucose ≥ 200 mg/dL after an oral glucose tolerance test, HbA1c ≥ 6.5%, using insulin or oral hypoglycemic agents, or self-reported physician-diagnosed diabetes [26]. The eGFR was calculated using the Chronic Kidney Disease Epidemiology Collaboration creatinine equation [27]. The NLR was derived from the complete blood count [24].

### Statistical analysis

According to the NHANES analytic guidelines [28], the appropriate sampling weights were used in the current analysis. The baseline characteristics of the included participants were presented as weighted means [standard error (SE)] for continuous variables and as frequency (weighted percentages) for categorical variables. The weighted linear regression (for continuous variables) or Rao-Scott chi-squared tests (for categorical variables) were applied to assess the differences in groups divided by HbA1c (tertiles).

In the current study, we used the weighted linear regression model to explore the effect size (β) and corresponding 95% confidence intervals (CIs) for AAC-24 score in relation to HbA1c levels. Additionally, weighted logistic regression models were performed to estimate odds ratios (ORs) and 95% CIs of HbA1c in relation to severe AAC. Sensitivity analyses were performed by using AAC-8 and severe AAC (AAC-8 ≥ 3) as the outcomes. Tests for linear trends across the HbA1c categories (tertiles) were conducted using an independent ordinal variable (0,1,2) in regression models. Moreover, subgroup analyses stratified by gender (male or female), age (< 60 or ≥ 60 years), BMI (< 25 or ≥ 25 kg/m^2^), smoker (yes or no), alcohol drinker (yes or no), hypertension (yes or no), diabetes (yes or no), and high cholesterol (yes or no) were also performed using multivariate regression models. Besides, an interaction term was added to test the heterogeneity of associations between the subgroups. The restricted cubic spline model was used for the dose-response analysis, using the cutoff value of lowest tertile of HbA1c (5.4%) as the reference. Diabetes status was regarded as a stratified factor in the models described above. Three models were performed in the regression models (Model 1: no covariates were adjusted; Model 2: adjusted for age, gender, BMI, race, education level, RIP, smoking status, alcohol drinking status, metabolic equivalent; Model 3: adjusted for covariates in model 2 plus SBP, TC, eGFR, total 25-hydroxyvitamin D, serum calcium, serum phosphorus, and NLR). Model 3 was used in the restricted cubic spline models and subgroup analyses.

The statistical analyses were performed using SAS 9.4 (SAS Institute Inc, Cary, NC, USA), except that the dose-response curve was drawn by R version 4.1.1 (R Foundation for Statistical Computing, Vienna, Austria). A two-sided *P* value < 0.05 was defined as statistical significance.

## Results

### Baseline characteristics

The baseline characteristics of the study population are presented in Table 1. The mean (SE) age of participants was 56.7 (0.3) years, and 51.8% of them were males. Compared with those in the lowest tertile group, participants in the higher tertile group were older, had higher BMI and FPG levels, had lower RIP and eGFR levels, and were more likely to have hypertension, high cholesterol, and diabetes. Additionally, the AAC-24 scores increased with the higher tertile of HbA1c. The average AAC-24 score was 1.3 (0.1) for the whole participants, and 0.7 (0.1), 1.2 (0.1), and 2.3 (0.2) for the lowest, middle, and highest tertile groups, respectively (*P* < 0.001). The prevalence of severe AAC was 6.7% overall, and increased with the higher tertiles of HbA1c as well (the lowest tertile, 2.7%; the middle tertile, 5.9%; the highest tertile,14.8%, *P* < 0.001).

**Table 1:** Weighted baseline characteristics of participants according to HbA1c level

	Overall (n = 1799)	Tertile 1 (≤ 5.4%) (n = 659)	Tertile 2 (5.4- 5.8%) (n = 546)	Tertile 3 (> 5.8%) (n = 594)	*P*-value
Age, year	56.7 ± 0.3	53.4 ± 0.4	58.0 ± 0.5	61.3 ± 0.3	< 0.001
Male, n (%)	933(51.8)	327(49.9)	282(52.4)	324(54.4)	0.216
BMI, kg/m^2^	28.2 ± 0.2	26.7 ± 0.2	28.7 ± 0.3	30.2 ± 0.3	< 0.001
Race, n (%)					< 0.001
Mexican American	220(6.4)	73(5.2)	65(6.4)	82(8.7)	
Other Hispanic	167(4.4)	58(3.9)	52(4.2)	57(5.5)	
Non-Hispanic White	827(73.0)	380(80.9)	252(73.3)	195(58.3)	
Non-Hispanic Black	352(9.8)	86(5.6)	103(9.7)	163(17.9)	
Other Race	233(6.3)	62(4.5)	74(6.4)	97(9.6)	
Education level, n (%)					< 0.001
Less than 9th grade	118(3.4)	23(2.1)	40(3.3)	55(5.9)	
9-11th grade	216(9.1)	70(7.6)	66(9.2)	80(11.7)	
High school graduate or GED	395(20.8)	140(18.5)	114(21.1)	141(24.7)	
Some college or AA degree	543(30.5)	200(29.0)	159(30.1)	184(34.0)	
College graduate or above	527(36.1)	226(42.8)	167(36.3)	134(23.7)	
RIP	3.29 ± 0.13	3.51 ± 0.16	3.23 ± 0.1	2.96 ± 0.1	< 0.001
Smoker, n (%)	967(54.9)	378(60.4)	283(50.3)	306(50.8)	0.011
Alcohol drinker, n (%)	459(19.2)	128(13.4)	134(19.0)	197(29.9)	< 0.001
Metabolic equivalent, MET-h/w	60.7 ± 2.4	59.9 ± 3.8	57.6 ± 4.2	66.2 ± 4.6	0.361
Hypertension, n (%)	805(41.0)	210(29.8)	229(40.4)	366(62.3)	< 0.001
SBP, mm Hg	124.7 ± 0.6	121.0 ± 0.8	125.6 ± 1.0	130.3 ± 1.0	< 0.001
DBP, mm Hg	71.8 ± 0.3	72.6 ± 0.3	71.7 ± 0.7	70.6 ± 0.5	0.009
High cholesterol, n (%)	686(38.7)	168(26.6)	195(41.7)	323(57.0)	< 0.001
TC, mg/dL	196.2 ± 1.2	197.5 ± 1.9	203.0 ± 2.0	185.2 ± 2.2	0.001
TG, mg/dL	119.8 ± 3.8	111.0 ± 6.4	120.9 ± 4.3	134.0 ± 6.3	0.005
HDL-C, mg/dL	55.5 ± 0.5	59.9 ± 0.7	54.8 ± 0.9	48.4 ± 1.1	< 0.001
LDL-C, mg/dL	117.4 ± 1.5	117.1 ± 2.4	121.8 ± 3.0	112.5 ± 2.6	0.373
Diabetes, n (%)	381(21.2)	19(2.1)	32(5.1)	330(55.6)	< 0.001
FPG, mg/dL	106.5 ± 1.0	96.5 ± 0.5	101.2 ± 0.8	130.7 ± 3.7	< 0.001
eGFR, mL/min/1.73 m^2^	85.2 ± 0.6	87.5 ± 0.9	85.6 ± 0.8	80.4 ± 0.7	< 0.001
Total 25-hydroxyvitamin D, nmol/L	75.2 ± 1.5	77.1 ± 2.4	75.6 ± 1.9	71.3 ± 1.7	0.068
Serum calcium, mmol/L	2.4 ± 0.0	2.4 ± 0.0	2.4 ± 0.0	2.4 ± 0.0	0.016
Serum phosphorus, mmol/L	1.2 ± 0.0	1.2 ± 0.0	1.2 ± 0.0	1.2 ± 0.0	0.756
NLR	2.3 ± 0.0	2.3 ± 0.1	2.3 ± 0.1	2.3 ± 0.1	0.539
AAC-24 score	1.3 ± 0.1	0.7 ± 0.1	1.2 ± 0.1	2.3 ± 0.2	< 0.001
Severe AAC, n (%)	141(6.7)	28(2.7)	34(5.9)	79(14.8)	< 0.001

Values are presented as weighted means ± standard error or frequency (weighted percentages) when appropriate. Abbreviations: BMI, body mass index; GED, general educational development; RIP, ratio of family income to poverty; SBP, systolic blood pressure; DBP, diastolic blood pressure; TC, total cholesterol; TG, triglyceride; LDL-C, low-density lipoprotein cholesterol; HDL-C, high-density lipoprotein cholesterol; TG, triglycerides; FPG, fasting plasma glucose; HbA1c, hemoglobin A1c; eGFR, estimated glomerular filtration rate; NLR, neutrophil-lymphocyte ratio; AAC, abdominal aortic calcification

### HbA1c level and AAC score

The weighted linear regression models showed a significant positive association between HbA1c and AAC-24 score (Table 2). When HbA1c was analyzed as a continuous variable, per unit (1%) increase in HbA1c level was associated with a 0.61 unit higher AAC-24 score (β = 0.61, 95% CI: 0.26–0.97) in the unadjusted model (model 1). This association remained statistically significant after adjusting for the covariates (model 2: β = 0.69, 95% CI: 0.29–1.08; model 3: β = 0.73, 95% CI: 0.30–1.16). When HbA1c was categorized into two groups (HbA1c < 6.5% and HbA1c ≥ 6.5%), the AAC-24 score of participants with HbA1c ≥ 6.5% was approximately 1.5 unit higher than that of those with HbA1c < 6.5% (β = 1.45, 95% CI: 0.33–2.58) in the fully adjusted model. Additionally, compared with the lowest tertile, the AAC-24 score of the middle and the highest tertiles was 0.40 and 1.48 units higher with a fully adjusted β (95%CI) of 0.40 (-0.38, 1.19) and 1.48 (0.56–2.39), respectively (*P* for trend < 0.003).

**Table 2:** Association of HbA1c level with AAC score

HbA1c level	β (95% CI)		
	Model 1	Model 2	Model 3
Continuous	0.61(0.26,0.97)**	0.69(0.29,1.08)**	0.73(0.30,1.16)**
Categories			
HbA1c < 6.5%	Reference	Reference	Reference
HbA1c ≥ 6.5%	1.25(0.26,2.23)*	1.41(0.33,2.49)*	1.45(0.33,2.58)*
Tertile 1	Reference	Reference	Reference
Tertile 2	0.57(-0.07,1.21)	0.37(-0.39,1.13)	0.40(-0.38,1.19)
Tertile 3	1.81(1.03,2.58)***	1.45(0.49,2.41)***	1.48(0.56,2.39)***
*P*-trend	< 0.001	0.002	0.003

Model 1: no covariates were adjusted;

Model 2: adjusted for age, gender, BMI, race, education level, RIP, smoking status, alcohol drinking status, metabolic equivalent;

Model 3: adjusted for covariates in model 2 plus SBP, TC, eGFR, total 25-hydroxyvitamin D, serum calcium, serum phosphorus, and NLR.

HbA1c: hemoglobin A1c; β: effect size; CI: confidence interval; BMI, body mass index; RIP, ratio of family income to poverty; SBP, systolic blood pressure; TC, total cholesterol; eGFR, estimated glomerular filtration rate; NLR, neutrophil-lymphocyte ratio; AAC, abdominal aortic calcification

*P < 0.05; **P < 0.01; ***P < 0.001

The AAC-8 score was used as the outcome of the sensitivity analysis. We obtained similar results with lower β, using the same models (Appendix Table 1). Per unit increase in HbA1c level was associated with a 0.27 unit higher AAC-8 score (β = 0.27, 95% CI: 0.11–0.42) in model 3. Compared with participants with HbA1c < 6.5%, the AAC-8 score of those with HbA1c ≥ 6.5% was 0.53 units higher with fully adjusted β (95%CI) of 0.53 (0.11–0.95). Moreover, compared with the lowest tertile, the AAC-8 score of the highest tertile was 0.58 units higher (β = 0.57, 95% CI: 0.23–0.91) in model 3.

### HbA1c level and severe AAC

The weighted logistic regression models indicated that higher HbA1c levels were associated with an elevated risk of severe AAC (Table 3). For every one unit increase (1%) in HbA1c level, the risk of severe AAC increased 46% in the unadjusted model (OR = 1.46, 95% CI: 1.26–1.68) and increased 63% in the fully adjusted model (OR = 1.63, 95% CI: 1.29–2.06). Compared with participants with HbA1c < 6.5%, the risk of severe AAC among those with HbA1c ≥ 6.5% were more than 3-fold higher. This result was stable in different models (model1: OR = 3.73, 95% CI: 2.05–6.78; model 2: OR = 3.46, 95% CI: 1.55–7.75; model 3: OR = 3.35, 95% CI: 1.35–8.25). When HbA1c was categorized into tertiles, participants in the middle and the highest tertiles of HbA1c showed a significant relative risk increase of developing severe AAC when compared with those in the lowest tertile, with unadjusted ORs (95% CIs) of 2.26 (1.46–3.49) and 6.22 (3.81–11.70) *(P* for trend < 0.001). This trend remained significant in model 3, with the lowest tertile of HbA1c as the reference, the fully adjusted ORs and 95% CIs of the middle and the highest tertiles categories were 1.55 (0.96–2.52) and 3.77 (1.57–9.09), respectively (*P* for trend < 0.006).

**Table 3:** Association of HbA1c level with severe AAC.

HbA1c level	OR (95% CI)		
	Model 1	Model 2	Model 3
Continuous	1.46(1.26,1.68)***	1.63(1.32,2.01)***	1.63(1.29,2.06)***
Categories			
HbA1c < 6.5%	Reference	Reference	Reference
HbA1c ≥ 6.5%	3.73(2.05,6.78)***	3.46(1.55,7.75)**	3.35(1.36,8.25)**
Tertile 1	Reference	Reference	Reference
Tertile 2	2.26(1.46,3.49)***	1.51(0.93,2.45)	1.55(0.96,2.52)
Tertile 3	6.22(3.81,11.70)***	3.69(1.56,8.77)**	3.77(1.57,9.09)***
*P*-trend	< 0.001	0.005	0.006

Model 1: no covariates were adjusted;

Model 2: adjusted for age, gender, BMI, race, education level, RIP, smoking status, alcohol drinking status, metabolic equivalent;

Model 3: adjusted for covariates in model 2 plus SBP, TC, eGFR, total 25-hydroxyvitamin D, serum calcium, serum phosphorus, and NLR.

HbA1c: hemoglobin A1c; OR: odds ratio; CI: confidence interval; BMI, body mass index; RIP, ratio of family income to poverty; SBP, systolic blood pressure; TC, total cholesterol; eGFR, estimated glomerular filtration rate; NLR, neutrophil-lymphocyte ratio; AAC, abdominal aortic calcification

**P < 0.01;***P < 0.001.

The results were similar in sensitivity analysis, considering an AAC-8 score of 3 or more as severe AAC (Appendix Table 2). Per unit increase in HbA1c level was associated with a 53% higher risk of severe AAC (OR = 1.53, 95% CI: 1.22–1.93) in model 3. Compared with participants with HbA1c < 6.5%, those with HbA1c ≥ 6.5% had a significantly higher risk of severe AAC (OR = 2.97, 95% CI: 1.31–6.74) in model 3. Additionally, compared with participants in the lowest tertile, those in the middle and highest tertiles of the HbA1c group were associated with a 40% (OR = 1.40, 95% CI: 0.73–2.71) and 203% (OR = 3.03, 95% CI: 1.21–7.64) higher risk of severe AAC, respectively (*P* for trend < 0.022).

### Subgroup analysis

**Fig. 2:**
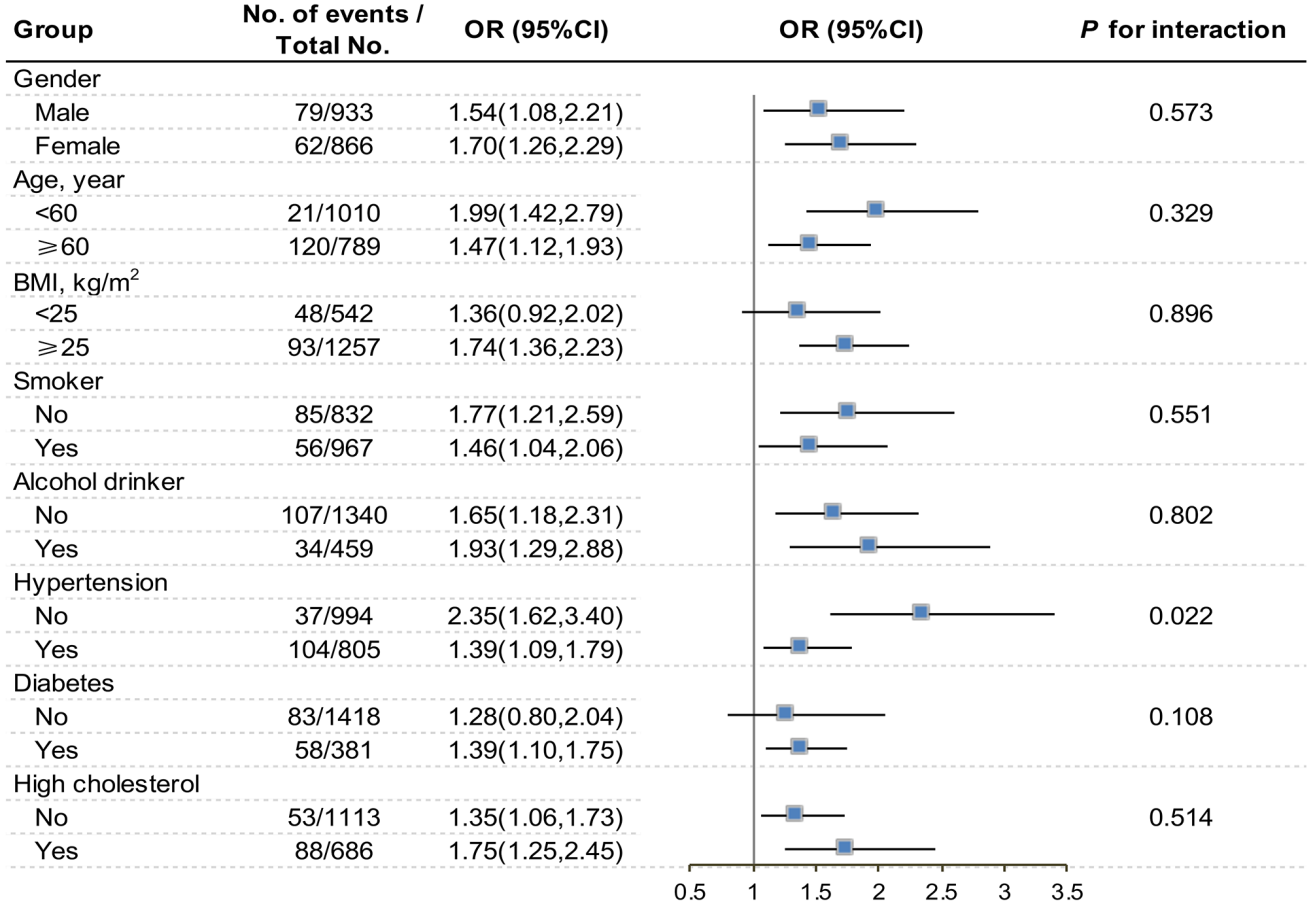
Subgroup analysis for the association between HbA1c level as a continuous variable and severe AAC. Odds ratio adjusted for variables in the Model 3 (age, gender, BMI, race, education level, RIP, smoking status, alcohol drinking status, metabolic equivalent, SBP, TC, eGFR, total 25-hydroxyvitamin D, serum calcium, serum phosphorus, and NLR) except the corresponding stratification variable OR: odds ratio; CI, confidence interval; BMI, body mass index; HbA1c, hemoglobin A1c

The subgroup analyses treating the HbA1c level as a continuous variable (per unit increase) are shown in Fig. 2 and Appendix Fig. 1. All associations were positive between HbA1c level and AAC (AAC score and severe AAC) among subgroups by gender, age, BMI, smoking status, alcohol drinker status, or health conditions (hypertension, diabetes, and high cholesterol). Notablely, the observed associations were not statistically significant when participants with diabetes were excluded. Besides, significant interactions effect between HbA1c and hypertension on severe AAC were observed (*P* for interaction = 0.022). The OR and 95% CI for each unit increase in HbA1c level for severe AAC was 2.35 (1.62–3.40) among normotensives vs. 1.39 (1.09–1.79) among hypertensives.

### Dose-response analysis

**Fig. 3:**
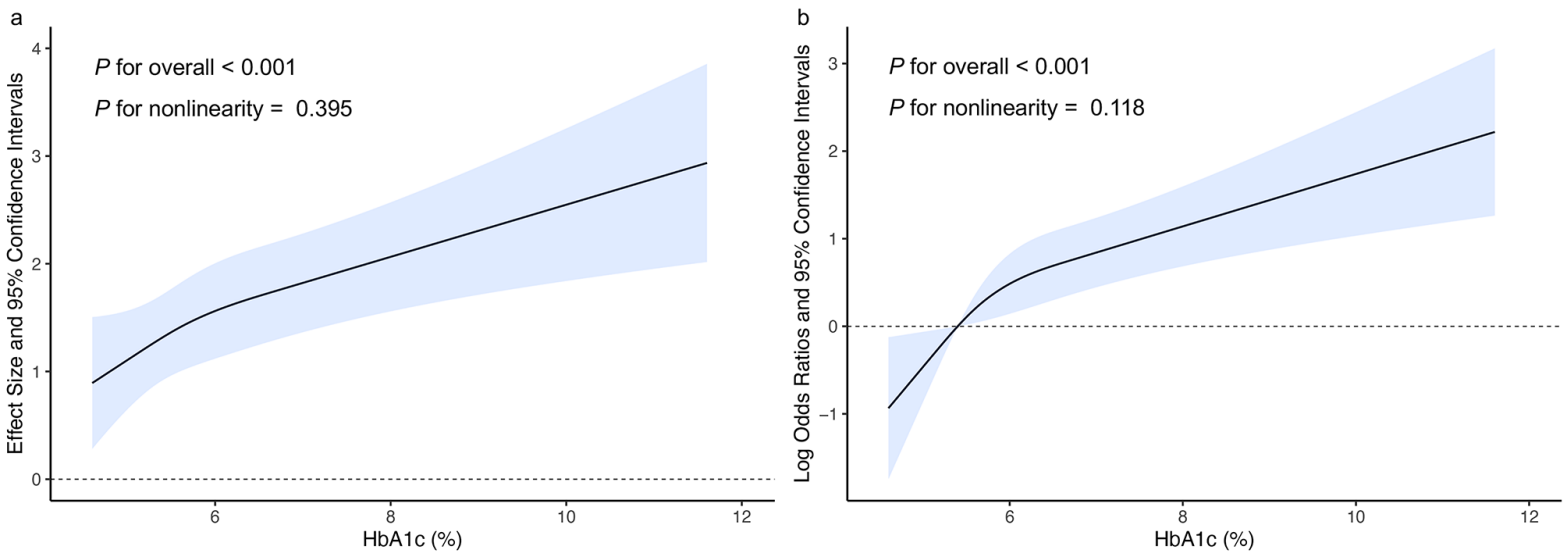
Dose-response relation between hemoglobin A1c level and AAC. **a**, Dose-response relation between hemoglobin A1c level and AAC-24 score; **b**, Dose-response relation between hemoglobin A1c level and severe AAC. The restricted cubic spline model was adjusted by age, gender, BMI, race, education level, RIP, smoking status, alcohol drinking status, metabolic equivalent, SBP, TC, eGFR, total 25-hydroxyvitamin D, serum calcium, serum phosphorus, and NLR. AAC, abdominal aortic calcification, HbA1c, hemoglobin A1c

As shown in Fig. 3, the dose-response analysis with a restricted cubic spline model showed a nearly linear relationship between the HbA1c level and AAC-24 score (*P* for nonlinearity = 0.395). Similarly, the HbA1c level was positively correlated with severe AAC (*P* for nonlinearity = 0.118) after adjustment for multiple potential covariates in Model 3.

## Discussion

In this nationally-representative cross-sectional survey, we comprehensively examined the effects of HbA1c level on AAC (AAC score and severe AAC). This study provided evidence that elevated HbA1c levels would increase the AAC score and the risk of severe AAC, resulting in nearly linear dose-response relationships after adjusting for multiple potential covariates. The directions of these relationships in different subgroups were consistent with that in the total population. Furthermore, the effect of HbA1c on severe AAC was more pronounced among normotensives than that among hypertensives. Since the levels of HbA1c is a “gold standard” for the assessment of glycemic control [9], our results emphasized the crucial effects of glycemic control on AC.

As a risk factor of CVD, AAC could be used as a tool for CVD risk assessment and could help clinicians identify individuals at high risk for clinical cardiovascular events and poorer long-term prognosis [7]. Diabetes is also a significant independent cardiovascular risk factor [8]. Previous studies have reported the association between AAC and diabetes, however, their results were ambiguous. Most studies observed that AAC was more prevalent in diabetes patients and diabetes was associated with a higher risk of AAC after multivariate analysis [23]. The diagnosis of diabetes depends on the glycemic traits (glucose and HbA1c levels) [29], whose effects on AAC are still rare. The relationships between glucose levels and AAC were explored preliminarily in subgroup analyses in two studies based on data from the NHANES study [26, 30]. The first study investigated the relationship between the triglyceride-glucose index and extensive AAC [26], and the second study revealed the link between metabolic syndrome and AAC [30]. The results of these studies showed that FPG was positively associated with AAC [26, 30]. However, these researches did not investigated the relationship between HbA1c levels and AAC, which was observed in the current study. We found one unit increase in HbA1c was associated with higher AAC scores (β = 0.73) and a higher risk of severe AAC (OR = 1.63). The JHS, examining the association of glycemic traits with AAC among African Americans, revealed that one SD increase in HbA1c (1.7%) led to a 0.33 unit increase in AAC scores [12]. It seems that the effect size in the current study was higher than that in JHS, which might be due to racial differences. Most participants were non-Hispanic whites (73.0%) in the current study, while all participants in the JHS were African Americans. Further research in large populations of different racial was needed to confirm the results.

To further explore whether the association between HbA1c and AAC was modified by gender, age, overweight, smoking, alcohol drinking, hypertension, diabetes, and high cholesterol, we conducted the subgroup analyses and tested the interaction effects between HbA1c and them. The protective effect of reducing HbA1c on AAC were found among both normotensives and hypertensives, and it was more pronounced among normotensives (*P* for interaction = 0.022), in other words, the protective effect of glycemic control on AAC was weakened when someone suffered from hypertension. Previous studies revealed that a vicious cycle existed between AC and hypertension due to the pathophysiological interplay between them [31, 32]. Particularly, hypertension is accompanied by remodeling of the arterial wall with changes in extracellular matrix composition and vascular cell phenotype modifications, which might lead to calcium deposition in the vascular wall. Moreover, calcium deposition could contribute to vascular stiffness and high BP [31]. The remodeling of the arterial wall and poor vascular conditions in hypertensives might weaken the protective effect of reducing HbA1c on AC. Additionally, no significant interactions were found between HbA1c and other factors, indicating that the association was consistent across gender, age, overweight, smoking, alcohol drinking, diabetes, and high cholesterol groups. Therefore, it is reasonable to speculate that almost every participant, especially normotensives, can reduce AAC through glycemic control.

The evidence on the dose-response relationships between HbA1c and AC were limited. Previous researchers have found that higher HbA1c was independently associated with advanced AAC scores, CAC risk, and CAC progression [10–12], however, they did not investigate the dose-response relationships between HbA1c and these outcomes. The current study showed a nearly linear dose-response relationships between HbA1c and AAC, which provided evidence for preventing AC through reducing HbA1c levels. Nevertheless, the Veterans Affairs Diabetes Trial have reported that participants with serious hypoglycemia had a higher risk of progression of CAC in patients with poorly controlled diabetes [33]. We did not found an increased risk of AAC when HbA1c was low, which may due to the differ proportion of diabetes patients in studies. Therefore, controlling blood glucose and HbA1c at appropriate levels might be beneficial for reducing the risk of AC. Further research in large population, especially diabetes patients, was needed to confirm the results and to determine an optimal HbA1c level for AC prevention.

Several potential mechanisms may contribute to the association between HbA1c and AAC. (1) Oxidative stress and inflammation: oscillating glucose and intracellular hyperglycemia have been reported to induce the overproduction of superoxide, which could decrease NO production, promote endothelial dysfunction, increase the expression of inflammation factors and adhesion factors, and formation of oxidized-low density lipoprotein [34]; (2) insulin resistance and hyperinsulinemia: greater long-term glycemic variability and excess sugar could induce the formation of advanced glycation end products [35, 36], which might contribute to the pathogenesis of insulin resistance [37]. Insulin resistance was associated with a cluster of metabolic abnormalities that promoted AC [38]; (3) osteogenic changes in vascular smooth muscle cells (VSMCs): transient hyperglycemia may induce alkaline phosphatase activation and osteogenic changes in VSMCs [39, 40]. These changes in vascular structure and function are common findings in the onset or progression of AC.

The current study investigated the effects of HbA1c on AAC based on data from a large and representative national survey the noninstitutionalized civilian resident population in the United States. A large representative sample, the rigorous study protocols, and standardized measurements of AAC and other covariates made our results representative and convictive. However, several limitations of the current study should be noted. First, the causal relationship between HbA1c on AAC could not be determined due to the cross-sectional design. Secondly, HbA1c could not capture short-term hypoglycaemia, hyperglycaemia and glycaemic fluctuations [41]. Further investigation is warranted to track the glucose profile using continuous glucose monitoring, and provide evidence for the effects of short-term glycemic control on AAC. Besides, since the subgroup analyses were exploratory and post hoc, we did not consider the multiple testing issue, which might increase the type I error rates when detecting the interaction effects. The specialized study design was required to confirm the association between HbA1c and AAC in different subgroups. In addition, a total of 1 258 participants with missing data on covariates were excluded, which might lead to selection bias. Compared with the excluded participants, those included in this study has a higher proportion of male, higher education levels, and slightly higher family income. Few studies have investigated the relationship of these socioeconomic statuses to AAC critically. Previous studies have reported that men had higher CAC scores than women [42], it is reasonable to speculate the existence of a gender difference in AAC [43]. Participants with higher education levels and higher family income receive higher quality care, have better health literacy, and a decreased number of CVD risk factors [44]. Indirectly, the AC status of these populations might be different from the total population. Those factors could affect the AAC scores and the occurrence of severe AAC. Therefore, further research in large populations was needed to confirm the results. Finally, AAC measurement was performed only in the population aged 40 years or above. Whether HbA1c influences AAC in adults aged < 40 years old warrants investigation.

## Conclusions

In summary, the current study advanced our understanding of the health benefits from lowering HbA1c levels in the population. We found elevated HbA1c levels would increase the AAC score and the risk of severe AAC with nearly linear dose-response relationships, which highlighted that long-term glycemic management is an important component of strategies for preventing AAC among all populations.

### Electronic supplementary material

Below is the link to the electronic supplementary material.

Appendix Table 1: Sensitivity analyses for association of HbA1c level with AAC score

Appendix Table 2: Sensitivity analyses for association of HbA1c level with severe AAC

Appendix Fig. 1: Subgroup analysis for the association between HbA1c level as a continuous variable and AAC score. Effect size adjusted for variables as the Model 3 (age, gender, BMI, race, education level, RIP, smoking status, alcohol drinking status, metabolic equivalent, SBP, TC, eGFR, total 25-hydroxyvitamin D, serum calcium, serum phosphorus, and NLR) except the corresponding stratification variable. β: effect size; CI, confidence interval; BMI, body mass index; HbA1c, hemoglobin A1c

## Data Availability

The data of the current study are available in the NHANES repository, https://www.cdc.gov/nchs/nhanes/.

## Abbreviations

AAC: Abdominal aortic calcification
AC: Arterial calcification
BMI: Body mass index
BP: Blood pressure
CAC: Coronary artery calcification
CI: Confidence interval
CVD: Cardiovascular disease
DBP: Diastolic blood pressure
eGFR: Estimated glomerular filtration rate
β: Effect size
FPG: Fasting plasma glucose
HbA1c: Hemoglobin A1c
JHS: Jackson Heart Study
MEC: Mobile examination center
NHANES: National Health and Nutrition Examination Survey
NLR: Neutrophil-lymphocyte ratio
OR: Odds ratio
RIP: Ratio of family income to poverty
SBP: Systolic blood pressure
SE: Standard error
TC: Total cholesterol
VSMC: Vascular smooth muscle cell
