# Supplementary material for: Association between hemoglobin A1c and abdominal aortic calcification: results from the National Health and Nutrition Examination Survey 2013–2014
Source: BMC Cardiovasc Disord. 2024 Jan 3;24:26. doi: 10.1186/s12872-023-03700-2 (PMC10765683; doi:10.1186/s12872-023-03700-2)
Supplement: Supplementary file 1 — Appendix Table 1: Sensitivity analyses for association of HbA1c level with AAC score [file 12872_2023_3700_MOESM1_ESM.docx]

**Appendix Table 1** Sensitivity analyses for association of HbA1c level with AAC score.

| HbA1c level | β (95% CI) | | |
| --- | --- | --- | --- |
|  | Model 1 | Model 2 | Model 3 |
| Continuous | 0.23(0.11,0.34)*** | 0.25(0.11,0.39)*** | 0.27(0.11,0.42)** |
| Categories |  |  |  |
| HbA1c<6.5% | Reference | Reference | Reference |
| HbA1c≥6.5% | 0.46(0.10,0.82)* | 0.52(0.11,0.93)* | 0.53(0.11,0.95)* |
| Tertile 1 | Reference | Reference | Reference |
| Tertile 2 | 0.22(-0.03,0.48) | 0.17(-0.14,0.47) | 0.18(-0.15,0.50) |
| Tertile 3 | 0.68(0.39,0.97)*** | 0.56(0.20,0.93)** | 0.57(0.23,0.91)*** |
| *P*-trend | <0.001 | 0.002 | 0.003 |

AAC-8 score was used for sensitivity analysis.

Model 1: no covariates were adjusted;

Model 2: adjusted for age, gender, BMI, race, education level, RIP, smoking status, alcohol drinking status, metabolic equivalent;

Model 3: adjusted for covariates in model 2 plus SBP, TC, eGFR, total 25-hydroxyvitamin D, serum calcium, serum phosphorus, and NLR.

HbA1c: hemoglobin A1c; β: effect size; CI: confidence interval; BMI, body mass index; RIP, ratio of family income to poverty; SBP, systolic blood pressure; TC, total cholesterol; eGFR, estimated glomerular filtration rate; NLR, neutrophil-lymphocyte ratio; AAC, abdominal aortic calcification.

**P* < 0.05 ***P* < 0.01; ****P* < 0.001;
