# Supplementary material for: Association between hemoglobin A1c and abdominal aortic calcification: results from the National Health and Nutrition Examination Survey 2013–2014
Source: BMC Cardiovasc Disord. 2024 Jan 3;24:26. doi: 10.1186/s12872-023-03700-2 (PMC10765683; doi:10.1186/s12872-023-03700-2)
Supplement: Supplementary file 2 — Appendix Table 2: Sensitivity analyses for association of HbA1c level with severe AAC [file 12872_2023_3700_MOESM2_ESM.docx]

**Appendix Table 2** Sensitivity analyses for association of HbA1c level with severe AAC.

| HbA1c level | OR (95% CI) | | |
| --- | --- | --- | --- |
|  | Model 1 | Model 2 | Model 3 |
| Continuous | 1.45(1.24,1.69)*** | 1.54(1.26,1.88)*** | 1.53(1.22,1.93)*** |
| Categories |  |  |  |
| HbA1c<6.5% | Reference | Reference | Reference |
| HbA1c≥6.5% | 3.56(2.01,6.33)*** | 3.25(1.54,6.89)*** | 2.97(1.31,6.74)** |
| Tertile 1 | Reference | Reference | Reference |
| Tertile 2 | 1.87(0.96,3.63) | 1.33(0.68,2.62) | 1.40(0.73,2.71) |
| Tertile 3 | 4.64(2.39,9.01)*** | 3.10(1.29,7.44)* | 3.03(1.21,7.64)* |
| *P*-trend | <0.001 | 0.013 | 0.022 |

AAC-8 score of 3 or more were considered as severe AAC in sensitivity analysis.

Model 1: no covariates were adjusted;

Model 2: adjusted for age, gender, BMI, race, education level, RIP, smoking status, alcohol drinking status, metabolic equivalent;

Model 3: adjusted for covariates in model 2 plus SBP, TC, eGFR, total 25-hydroxyvitamin D, serum calcium, serum phosphorus, and NLR.

HbA1c: hemoglobin A1c; OR: odds ratio; CI: confidence interval; BMI, body mass index; RIP, ratio of family income to poverty; SBP, systolic blood pressure; TC, total cholesterol; eGFR, estimated glomerular filtration rate; NLR, neutrophil-lymphocyte ratio; AAC, abdominal aortic calcification.

**P* < 0.05; ***P* < 0.01; ****P* < 0.001.
