# Supplementary material for: Association between hemoglobin A1c and abdominal aortic calcification: results from the National Health and Nutrition Examination Survey 2013–2014
Source: BMC Cardiovasc Disord. 2024 Jan 3;24:26. doi: 10.1186/s12872-023-03700-2 (PMC10765683; doi:10.1186/s12872-023-03700-2)
Supplement: Supplementary file 3 — Appendix Fig. 1: Subgroup analysis for the association between HbA1c level as a continuous variable and AAC score. Effect size adjusted for variables as the Model 3 (age, gender, BMI, race, education level, RIP, smoking status, alcohol drinking status, metabolic equivalent, SBP, TC, eGFR, total 25-hydroxyvitamin D, serum calcium, serum phosphorus, and NLR) except the corresponding stratification variable. β: effect size; CI, confidence interval; BMI, body mass index; HbA1c, hemoglobin A1c [file 12872_2023_3700_MOESM3_ESM.docx]

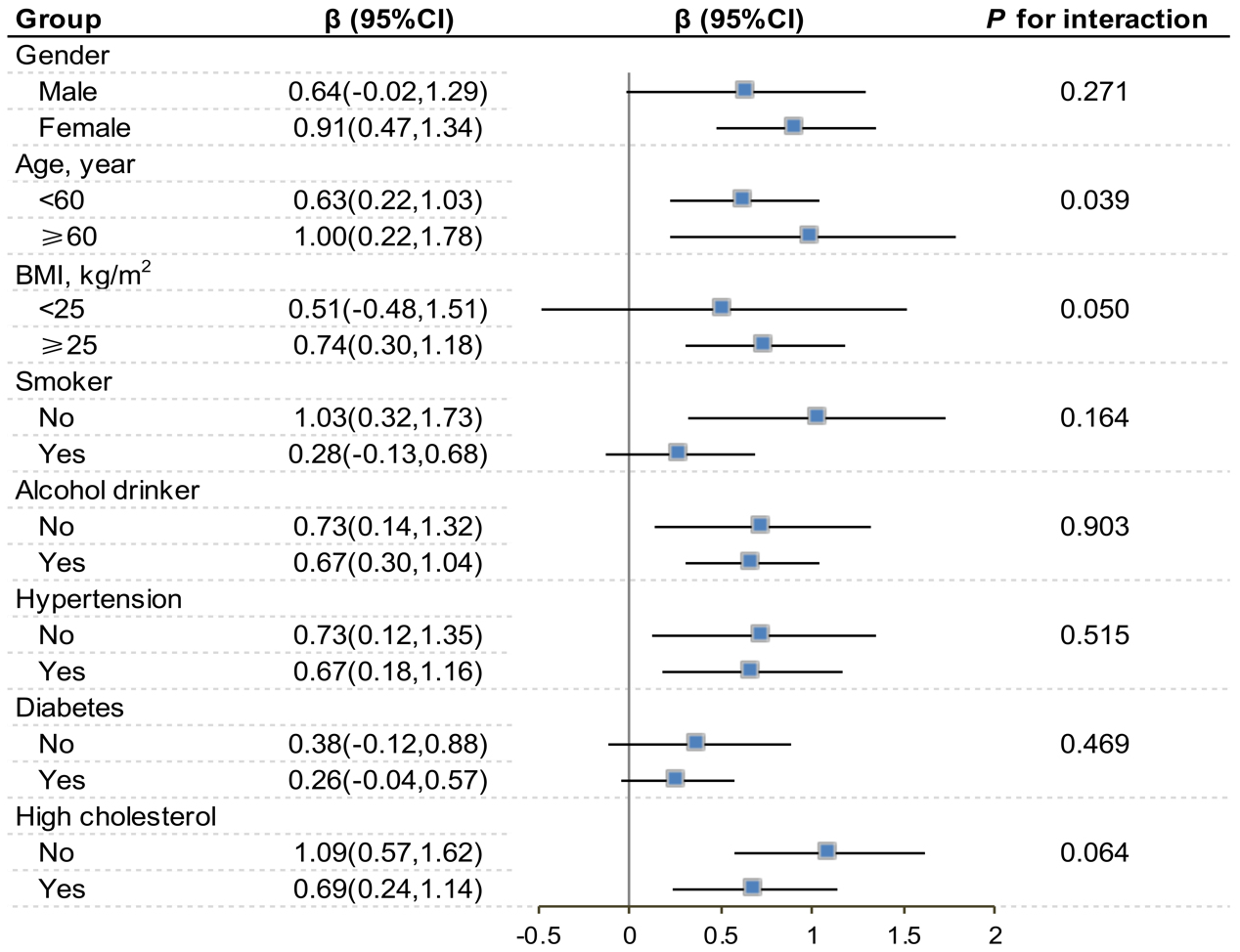


**Appendix Fig. 1** Subgroup analysis for the association between HbA1c level as a continuous variable and AAC score.

Effect size adjusted for variables as the Model 3 (age, gender, BMI, race, education level, RIP, smoking status, alcohol drinking status, metabolic equivalent, SBP, TC, eGFR, total 25-hydroxyvitamin D, serum calcium, serum phosphorus, and NLR) except the corresponding stratification variable.

β: effect size; CI, confidence interval; BMI, body mass index; HbA1c, hemoglobin A1c.
